# Functional interplay between antagonistic bacteria and *Rhizoctonia solani* in the tomato plant rhizosphere

**DOI:** 10.3389/fmicb.2022.990850

**Published:** 2022-09-26

**Authors:** Manoj Kumar Solanki, Anjali Chandrol Solanki, Shalini Rai, Supriya Srivastava, Brijendra Kumar Kashyap, Praveen Kumar Divvela, Sudheer Kumar, Mahesh S. Yandigeri, Prem Lal Kashyap, Alok Kumar Shrivastava, Baber Ali, Shahid Khan, Mariusz Jaremko, Kamal Ahmad Qureshi

**Affiliations:** ^1^Faculty of Natural Sciences, Plant Cytogenetics and Molecular Biology Group, Institute of Biology, Biotechnology and Environmental Protection, University of Silesia in Katowice, Katowice, Poland; ^2^Department of Agriculture Science, Mansarovar Global University, Bhopal, MP, India; ^3^Department of Biotechnology, Society of Higher Education and Practical Application (SHEPA), Varanasi, UP, India; ^4^Department of Health Informatics, College of Public Health and Health Informatics, Qassim University, Al Bukayriyah, Saudi Arabia; ^5^Department of Biotechnology Engineering, Institute of Engineering and Technology, Bundelkhand University, Jhansi, UP, India; ^6^Contec Global Agro Limited, Abuja, Nigeria; ^7^Indian Institute of Wheat and Barley Research (ICAR), Karnal, HR, India; ^8^National Bureau of Agricultural Insect Resources (ICAR), Bengaluru, KA, India; ^9^National Bureau of Agriculturally Important Microorganisms (ICAR), Kusmaur, UP, India; ^10^Department of Plant Sciences, Quaid-i-Azam University, Islamabad, Pakistan; ^11^Department of Agriculture, University of Swabi, Swabi, Pakistan; ^12^Department of Plant Breeding and Genetics, University of Agriculture Swat, Peshawar, Pakistan; ^13^Smart-Health Initiative (SHI) and Red Sea Research Center (RSRC), Division of Biological and Environmental Sciences and Engineering (BESE), King Abdullah University of Science and Technology (KAUST), Thuwal, Saudi Arabia; ^14^Department of Pharmaceutics, Unaizah College of Pharmacy, Qassim University, Unaizah, Saudi Arabia

**Keywords:** *pseudomonas*, *bacillus*, BIOLOG, community-level physiological profile, disease incidence

## Abstract

Microbial interactions with plant roots play an imperial role in tomato plant growth and defense against the *Rhizoctonia solani*. This study performed a field experiment with two antagonistic bacteria (*Pseudomonas* and *Bacillus*) inoculated in healthy and *Rhizoctonia solani* treated soil in tomato rhizosphere to understand the metabolic pattern and microbial function during plant disease suppression. In the present study, we assessed soil and microbial enzymes, bacterial and fungal cell forming unit (CFU), and carbon utilization profiling through Bio-Eco plates of rhizoplane samples. Antagonist bacteria and pathogen interaction significantly (*p* < 0.05) influenced the bacterial count, soil enzymes (chitinase and glucanase), and bacterial function (siderophore and chitinase production). These results indicated that these variables had an imperial role in disease suppression during plant development. Furthermore, the metabolic profiling showed that carbon source utilization enhanced under fruit development and ripening stages. These results suggested that carbon sources were essential in plant/pathogen/antagonist interaction. Substrates like β-methyl-D-glucoside, D-mannitol, D-galacturonic acid, N-acetyl-D-glucosamine, and phenylethylamine strongly connect with the suppuration of root rot disease. These carbon sources may help to propagate a healthy microbial community to reduce the pathogen invasion in the plant root system, and these carbon sources can be stimulators of antagonists against pathogens in the future.

## Introduction

Soil is a reservoir of microbial activities that are driven through numerous signaling molecules that helps them to sustain in harsh environments (Haldar and Sengupta, 2015; Jacoby et al., 2017). Rhizospheric microbes are the significant players in nutrient cycling that play an essential role in plant development (Bulgarelli et al., 2013; Francioli et al., 2016; Müller et al., 2016). The plant rhizosphere contains beneficial and pathogenic microbes competing for nutrients and space (Raaijmakers et al., 2009; Adesemoye et al., 2009; Beans, 2017; Li et al., 2021). Tomato root rot caused by sclerotia forming fungus *Rhizoctonia solani*, is a highly destructive disease that severely affects crop development and yield (Patil and Solanki, 2016b). To control *R. solani*, chemical fungicides must be applied, creating many environmental problems (Le Cointe et al., 2016). In the pathogen-treated rhizosphere, many physicochemical and biological processes are mechanized surrounding the plant root through the microbes (Reva et al., 2004; Raaijmakers et al., 2009; Mhlongo et al., 2018; Berendsen et al., 2018; Pascale et al., 2020a; Tahat et al., 2020). Moreover, antagonistic microbes’ application against soilborne plant pathogens is one of the most numerous anthropogenic activities that reform soil health and plant defense (Solanki et al., 2012b,^2014^; Yin et al., 2013; Abbas et al., 2019; Lahlali et al., 2022). The role of the different carbon substrates in multitrophic interaction (plant/antagonist/pathogen) needs to be studied in depth to improve plant disease management techniques.

A wide range of natural bacterial antagonists are utilized as biocontrol agents against seed and soilborne plant pathogens (Patil and Solanki, 2016a; Solanki et al., 2019, 2020). *Bacillus* and *Pseudomonas* genera are the most prevalent biological agents (Solanki et al., 2014, 2015; Cao et al., 2018; Abbas et al., 2019). Most bacterial antagonists create an obliging interaction with plant roots that can modulate by the selective pressure of changing environment (Bais et al., 2006; Falardeau et al., 2013). For example, it is well known that pathogens influence the production and diffusion of root exudates (Guo et al., 2015; Hoysted et al., 2018; Pascale et al., 2020a; Li et al., 2021). Interestingly, plant-pathogen associations are modulated through native microbial communities during infestation and resistance (Chiu et al., 2017; Stevens et al., 2021; Dubey et al., 2022). Root exudates generally release carbohydrates, carboxylic acids, amino acids, sugars, phenolics, proteins, and allelochemicals (Moe, 2013; Guo et al., 2015; Olanrewaju et al., 2019; Scavo et al., 2019). It indirectly regulates the controls of the biotic and abiotic processes by shaping the microbial communities that can metabolize the substrates and nutrients (Vacheron et al., 2013; Antoniou et al., 2017; Bakker et al., 2018; Lladó et al., 2018). Different sites of plant roots have been characterized for releasing specific exudates, such as the sub-apical zone, root-hair zone, and emerging areas of secondary ramifications (Bais et al., 2006), and these areas play a vital role in plant-plant and plant-microbes interaction (Vacheron et al., 2013; Khashi et al., 2019). Exudates are a suitable source of carbon (and possibly nitrogen) and energy for root-associated microbes (Haldar and Sengupta, 2015; Sun et al., 2019; Canarini et al., 2019). The microbial communities that metabolize these carbon sources survive easily in the root zone (Compant et al., 2010; Pascale et al., 2020b).

Subsequently, essential soil functions are crosslinked with rhizospheric microbial activities such as iron chelation, phosphate solubilization, nitrogen fixation, antagonism, and bioremediation (Patil and Solanki, 2016a; Li et al., 2018). To identify the metabolic potential of antagonistic microbes through BIOLOG ECO plates that contain 31 various carbon sources have been used (Di Bonito and Biagiotti, 2021; Németh et al., 2021; Moreno et al., 2021; Petkova et al., 2022; Koner et al., 2022). Nine of the 31 substrates of ECO plates are known as components of exudates of plant roots (Insam, 1997). The Community level physiological profiles (CLPP) approach has often been used to assess the functional diversity that is influenced by microbes or other environmental practices (Iliev et al., 2021; Koner et al., 2021; Aleksova et al., 2021; Sneha et al., 2021; Jacobs-Hoffman and Hills, 2021; Kumar et al., 2021; Dubey et al., 2022).

The plant pathogenic fungi can infect plants at any developmental stage, but the infection is particularly favored when plants are weakened due to nutritional disorders in response to climatic pressure (Divon and Fluhr, 2007; Velásquez et al., 2018). Therefore, the present study focused on a few essential questions that need to be answered: What relationship is undergoing between native microbial responses and antagonists? What are the significant metabolic linkages in pathogen inhibition by antagonists? What is the significance of different kinds of substrates in disease inhibition? Therefore, we hypothesize that rhizodeposition influences microbial activity and diversity indices during plant development. To unlock the above queries, two biocontrol agents, *Pseudomonas fluorescens* MPF47 (Solanki et al., 2014) and *Bacillus velezensis* MB101 (heterotypic synonym of *B. amyloliquefaciens*) (Solanki et al., 2012a,^2019^) were used as an antagonist against *R. solani* in this study and an filed experiment was performed. Next, BIOLOG ECO plates have been used to assess community-level physiological profiles of different treatments with and without pathogen. Soil microbial dynamics and enzymes and bacterial activities have been assessed to see the links between substrate diversity and microbial activities.

## Materials and methods

### Antagonist inoculum preparation

Active culture (1 mL) of strains (*Pseudomonas fluorescens* MPF47 and *Bacillus* velezensis MB101) was inoculated in a 500 mL flask containing 250 mL of nutrient broth (HiMedia, India) on a rotary shaker (120 rpm) at 28 ± 2°C for 24 h. Bacterial cells were pelleted by centrifugation 6,000 × *g* for 10 min (Sigma 3K30 centrifuge, Germany) and suspended (10^8^ cells mL^–1^) in 100 mL sterile solution (2.0% polyvinyl pyrrolidine (PVP), 1.5% polyethylene glycol (PEG) and 2.5% glycerol), mixed aseptically and stored in sterile glass bottles for treatment.

### Plant material and experiment setup

Surface sterilized tomato (*Lycopersicon esculentum* Mill.) seeds of a native variety were grown in seedling trays that were treated with three different kinds of treatments: 1) antagonist MPF47 (1 × 10^8^ cells ml) inoculum 10 ml kg^–1^, 2) antagonist MB101 (1 × 10^8^ cells ml) inoculum 10 ml kg^–1^ and 3) sterilized liquid suspension without bacteria. All trays were incubated for four weeks under a glasshouse (RH 80%, 12:12 h 28°C day, and 22°C night). After four weeks, seedlings were again treated with the same bacterial formulations using the root dipping method. All treated seedlings were air dried and manually transplanted into the experimental field. The soil had the following characters: clay 22.4%; bulk density 48.2 g/cm^3^; sand 57%; silt 24.1%; water holding capacity 67.28%; pH 6.02; EC_*e*_ 1.40dS m^–1^; organic matter 2.94%; organic C 138.02 kg ha^–1^; total N 94 kg ha^–1^; P 10.21 kg ha^–1^; Zn 0.510 mg kg^–1^; Mn 22.11 mg kg^–1^; Fe 15.21 mg kg^–1^; Cu 1.8 mg kg^–1^; and S 9.1 mg kg^–1^ and microbial density bacteria (7.10 log CFU g^–1^ soil), and fungus (5.50 log CFU g^–1^ soil). *R. solani* culture was grown in pearl millet seeds under aseptic conditions, according to Solanki et al. (2011). Pathogen-sick plots were prepared before transplantation by inoculating the pearl millet culture of *R. solani*, according to Solanki et al. (2019). A healthy plot mixed with the autoclaved pearl millet culture of *R. solani* served as control. Bacterial antagonist-treated seedlings were transplanted in field soil by the following treatments: (T1) *Pseudomonas* alone, (T2) *Bacillus* alone, (T3) healthy control (autoclaved liquid suspension without bacteria), (T4) antagonist *Pseudomonas* + *R. solani*, (T5) *Bacillus* + *R. solani*, and (T6) *R. solani* alone with autoclaved liquid suspension without bacteria. Each treatment was replicated three times, and treatments were arranged in field plots (4 × 4 m) comprising five rows per plot and five plants per row in a completely randomized block design. All the agronomic practices such as hand weeding and fertilizers ((120 kg ha^–1^ nitrogen (N), 50 kg ha^–1^ phosphorus (P_2_O_5_), and 50 kg ha^–1^ potash (K_2_O)) at the same rate for all the treatments was followed.

### Plant parameters

All treated tomato seedlings were removed from the soil at different growth stages, and roots were washed with sterile distilled water. The disease index (DI%) was calculated according to Solanki et al. (2011). Twenty randomly selected plants from each plot were carefully uprooted after 110 days of transplanting and used for measurement of root length (cm), plant height (cm), total plant biomass without fruits (g), and fruit biomass (g).

### Soil microbial activity and enzymes

**FIGURE 1:**
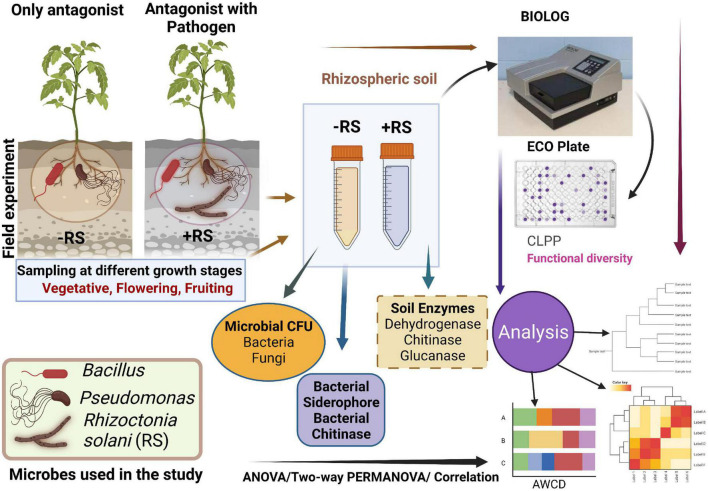
Schematic representation of the present study. CFU-Colony forming unit, CLPP- community-level physiological profiling.

Rhizosphere soil sampling was performed from each treatment, and a composite soil sample was collected and analyzed according to Figure 1. Samplings were conducted on three occasions in accord to stages of the plant on a different days after transplantation (DAT) at different growth stages; Stage 1 = vegetative stage (25 DAT), stage 2 = fruit development stage (60 DAT), stage 3 = fruit ripening and harvesting (110 DAT). All soil samples were sieved to 2 mm particle size and used immediately, as presented in Figure 1. The total active microbial biomass was enumerated from soils by the serial dilution method. Different agar media were employed for the isolation and enumeration of bacterial and fungal biomass. The population of bacteria was enumerated on nutrient agar (HiMedia, India), and the total fungal biomass population was isolated using potato dextrose agar (HiMedia, India) supplemented with antibacterial antibiotics streptomycin (500 μg mL^–1^) and chloramphenicol (25 μg mL^–1^). Moreover, three soil enzymes were assessed: dehydrogenase, chitinase, and β-1, 3 Glucanase. Soil dehydrogenase activity was evaluated by the method of Singh and Singh (2005). Soil chitinase was determined using the modified method of Trotta et al. (1996). β-1, 3 Glucanase was assayed by a modified protocol using laminarin as a substrate, according to Lethbridge et al. (1978). Isolated soil bacteria were purified and used for the chitinase and siderophore production assays. Bacterial siderophore production was detected using the chrome azurol S (CAS) method according to Solanki et al. (2014), and chitinase enzyme production was determined according to Solanki et al. (2012b) and. All screening experiments were repeated three times.

### BIOLOG ECO plate assays and analysis

BIOLOG ECO plates (Biolog, Inc., Hayward, CA, United States) were used to determine substrate utilization by the microbial community from the rhizosphere soil of the tomato plant. The soil from each composite sample was homogenized, and 5 g was used for the analysis. Triplicate 5 g fresh samples were suspended in 45 mL sterile saline solution (NaCl, 0.85%) with 3 mm glass beads (5 g) on a rotary shaker at 220 rpm for 30 min at 25°C. The suspensions were allowed to settle for 5 min, and then 10-fold diluted samples were prepared, and 125 μL aliquots of dilutions were added to each plate well. The absorbance (590 nm) was read using an automated BIOLOG Microplate TM Reader, and data were collected using the MicroLog 4.01 software. The plates were then sealed inside a plastic bag, incubated at 25°C in darkness, and read every 24 h for seven days. To analyze the BIOLOG reader data, the absorption value of the control well was subtracted from each substrate absorption value, while substrates with negative values were considered non-oxidized. The average well color development (AWCD), calculated as the average optical density across all wells per plate, was used to indicate general microbial activity (Garland and Mills, 1991; Garland, 2006; Grzadziel et al., 2019). AWCD value at 120 h was used to describe the difference in rhizoplane microbial activities among the different treatments. AWCD = Σ(C−R)/n C-reading of the well OD; R-reading of the control well OD; n-the number of substrates on an EcoPlate™ (31).

### Statistical analysis

Microbial siderophore and chitinase data were represented through a bar plot. Mean catabolic activity and mean of AWCD were calculated from data of all three developmental stages. Shannon, McIntosh, Simpson diversity indices, and evenness were estimated using BIOLOG™ ECO plates and generated box plot. Boxplots of the mean, standard deviation (SD) and boxes include the interquartile range and the line inside the box represents group median values. The whiskers bars indicate the minimum and maximum values excluding outliers (circles). The notch displays the 95% confidence interval around the median. Principal coordinate analysis (PCoA) was performed on the BIOLOG™ ECO plate data to characterize the microbial response in different growth stages. A response heatmap was generated by the TB tools (Chen et al., 2020). Moreover, individual values of optical density (OD) were grouped into six categories, namely, amines and amides, amino acids, carboxylic and acetic acids, carbohydrates, acid derivatives of carbohydrates, and polymers. BIOLOG data, along with values concerning diversity parameters, soil parameters, and plant parameters, were analyzed through a Two-way PERMANOVA (Permutation N-9999), with treatments and time (growth stages) as grouping variables of the healthy (-RS) and pathogen-treated soil (+ RS). For additional multiple *post hoc* comparisons, a Duncan’s Multiple Range Test (DMRT) was used for ANOVA analysis by using IBM SPSS Statistics, version 25, IBM Corp., Armonk, NY, United States. Moreover, the correlation analysis between the substrate and all plant and soil parameters was performed using Past3 software. A correlation heatmap was generated using TB tools.

## Results

### Plant growth, biomass, and disease incidence

Treatment T1 (*Pseudomonas*) and T2 (*Bacillus*) enhanced the root and shoot length up to 1.16-1.29 and 1.25-1.34 times higher than healthy control (T3), respectively. However, in pathogen-treated soil, *Pseudomonas* + RS (T4) and *Bacillus* + RS (T5) increased root length by 2.36- 2.55 times and plant shoot length by 2.05- 2.45 times as compared to pathogen control (T6) (Supplementary Figure 1A). *Bacillus*-treated plants showed higher plant dry biomass and fruit biomass in healthy and pathogen-treated soil (Supplementary Figure 1B). The symptoms of *Rhizoctonia* root rot appeared in stage 2 and stage 3 (Supplementary Figure 1C). The disease indices estimated at stages 2 and 3 were significantly higher in *R. solani* (T6) compared to both antagonists with pathogen (T4 and T5). A significant disease reduction resulted in both antagonists over the pathogen control (Supplementary Figure 1D).

### Soil microbial activity and different enzymes

**FIGURE 2:**
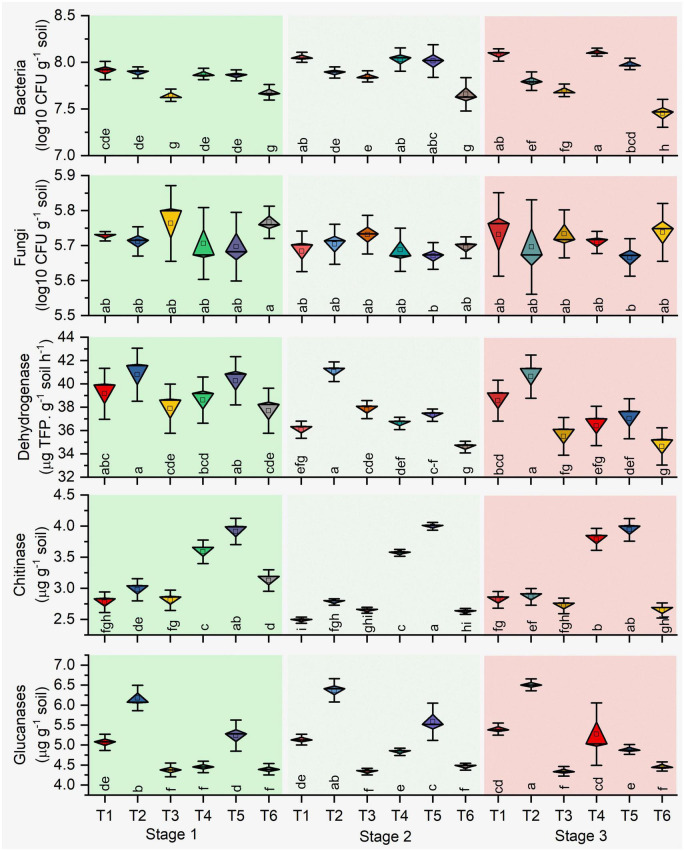
Impact of pathogen and antagonist treated soil on the microbial count and soil enzymes. Treatments: (T1) *Pseudomonas* alone, (T2) *Bacillus* alone, (T3) healthy control (autoclaved liquid suspension without bacteria), (T4) antagonist *Pseudomonas* + *R. solani*, (T5) *Bacillus* + *R. solani*, and (T6) *R. solani* alone with autoclaved liquid suspension without bacteria. Stage 1 (vegetative stage), Stage 2 (flowering stage), and Stage 3 (fruit ripening stage). Mean values (n = 3) in the same column followed by the same letter(s) are not significantly different at (*P* < 0.05) according to the DMRT test.

The microbial count of rhizosphere soil represents the soil biology, and total bacterial counts and bacterial CFU increased significantly (*P* ≤ 0.05) in stage 2 in all treatments. Higher CFU resulted in antagonist treatments (T1, T2, T4, and T5) over the plant growth. Besides, different growth stages determined the lower bacterial count resulting in the pathogen-treated soil samples (Figure 2). Significant effects on bacterial CFU were observed for antagonists (*p* = 0.001) and growth stage (*p* < 0.001) and their interaction (*p* = 0.001) in healthy soil. Similarly, pathogen-treated soil bacterial CFU were observed for antagonists (*p* = 0.054) and growth stage (*p* < 0.001) and their interaction (*p* = 0.002) (Table 1). Total fungal count significantly (*p* < 0.05) impacted with the antagonist (T4 and T5) in the pathogen-treated soil, and it was least affected in the healthy soil treatments (T1, T2, and T3). However, *Bacillus* (T2 and T5) treated plants reduced the fungal counts in the healthy and pathogen-treated soil (Figure 2). Compared to other treatments, a higher fungal population was recorded with pathogen-treated (T6) and healthy controls (T3) (Figure 2). In the case of soil enzymes, healthy soil treatments (T1, T2, and T3) showed higher dehydrogenase activity than *R. solani* treated soil samples (T4, T5, and T6) during plant growth. Both bacterial antagonist samples have higher biological activity in the absence of pathogen. Besides, soil chitinase activity was found to be strong in pathogen-treated soil, and both antagonists treatments (T4 and T5) samples showed higher chitinase activity in stages 1 and 3. For soil glucanases, higher activity was revealed in healthy soil treatments compared to pathogen-treated soil (Figure 2). Significant effects on the two enzymes chitinase and glucanase were observed for antagonist treatments (*p* < 0.06) and growth stage (*p* < 0.001) and their interaction (*p* < 0.01) in pathogen-treated soil (Table 1).

**TABLE 1:** The *P*-values of PERMANOVA for soil parameters, microbial count, diversity indices, and different classes of AWCD rate in *R. solani* infected and healthy soil.

Parameters		Healthy soil (-RS)			Pathogen-infected soil (+ RS)		
		T	GS	T × GS	T	GS	T × GS
CFU	Bacterial	0.001**	0.00***	0.001**	0.054	0.00***	0.002**
	Fungal	0.566	0.321	0.898	0.210	0.051	0.762
Soil enzymes	Dehydrogenase	0.118	0.00***	0.017*	0.00***	0.00***	0.846
	Chitinase	0.00***	0.001**	0.113	0.024*	0.00***	0.00***
	Glucanase	0.012*	0.00***	0.114	0.065	0.00***	0.003**
Bacterial	Siderophore (%)	0.001**	0.00***	0.122	0.00***	0.00***	0.001**
	Chitinase (%)	0.00***	0.00***	0.021*	0.00***	0.00***	0.008**
Metabolic responce	AWCD (120 h)	0.003**	0.090	0.520	0.00***	0.003**	0.012*
	CMD	0.031*	0.00***	0.366	0.00***	0.024*	0.199
Diversity indices	Shannon index	0.146	0.989	0.465	0.00***	0.001**	0.00***
	Simpson index	0.00***	0.011*	0.069	0.003**	0.398	0.134
	McIntosh index	0.003**	0.020*	0.823	0.00***	0.018*	0.037*
	Substrate richness	0.350	0.661	0.189	0.00***	0.00***	0.020*
	Substrate evenness	0.292	0.946	0.694	0.001**	0.001**	0.00***
Substracte classes	Amines/amides	0.018*	0.011*	0.100	0.026*	0.025*	0.089
	Amino acids	0.132	0.020*	0.209	0.00***	0.199	0.730
	Carbohydrates	0.322	0.015*	0.036*	0.00***	0.027*	0.040*
	Acids derived from carbohydrate	0.218	0.002**	0.059	0.084	0.166	0.138
	Carboxylic & acetic acids	0.057	0.285	0.010**	0.00***	0.001**	0.415
	Polymers	0.108	0.664	0.608	0.001**	0.241	0.072

Average well color development (AWCD), community metabolic diversity (CMD), *Rhizoctonia solani* (RS), Colony forming unit (CFU); Bacterial treatments (T): *Pseudomonas* and *Bacillus*; Growth stages (GS): different growth stages of tomato; Significance level *p < 0.05, **p < 0.01 and ***p < 0.001.

### Bacterial frequency in siderophore and chitinase production

**FIGURE 3:**
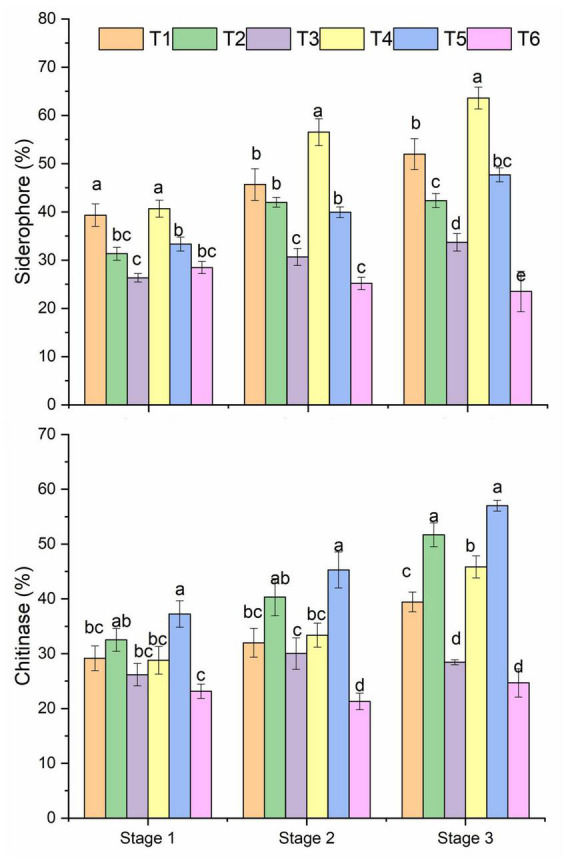
Microbial siderophore and chitinase frequency of bacteria isolated from different treatments. Mean values (*n* = 3) in the same column followed by the same letter(s) are not significantly different at (*P* < 0.05) according to the DMRT test. Treatment details as Figure 2.

All treatments showed a differential pattern of siderophore-producing bacteria in the healthy and *R. solani* treated soil (Figure 3 and Table 1). A higher frequency of siderophore bacteria was found in *Pseudomonas* + RS (T4) and only *Pseudomonas* (T1), followed by only *Bacillus* (T2). The stage 3 soil samples showed a higher frequency among the three growth stages. Moreover, a treatment-wise comparison revealed that *Pseudomonas* + RS treated soil samples have a higher number of siderophore-producing bacterial communities (Figure 3). A differential pattern of chitinase activity was also revealed among the treatments. Bacterial antagonists (T5 and T2) showed higher chitinase-producing bacteria frequency in all growth stages, especially in stage 3 (Figure 3). Results from a two-way PERMANOVA showed significant (*p* < 0.01) interaction of pathogen in siderophore and chitinase-producing bacteria frequency (Table 1).

### Community-level physiological profile

The AWCD, as a measure of the total microbial activity, generally followed the different patterns with treatments. The microbial activities tended to increase in the vegetative stage and changed gradually. We recorded maximum AWCD response in *Bacillus* (T2) and *Pseudomonas* + RS (T4) treatments in stage 1 and stage 3 (Supplementary Figure 2). The pathogen-treated soil has lower AWCD responses as compared to healthy soil. In case of CMD response, antagonistic bacteria show higher activity (Supplementary Figure 2). The *P*-values for the AWCD parameter presented in Table 1 showed that antagonists and pathogen treatment significantly (*p* = 0.003) affect the microbial metabolic activity. The AWCD values showed that pathogen-treated soil significantly changed during the plant growth than the healthy soil. In the case of CMD, no significant interaction was found between treatments and time (Table 1). Microbial responses of antagonists showed stability with pathogen-treated soil alone up to stage 3. PCA soil showed that *Psudomonase* (T1) grouped well in stage 2 and stage 3 but detached in stage 1 in healthy soil (Supplementary Figure 3A). *Bacillus* (T2) bacteria showed grouping in stage 1 and stage 3 in healthy soil. However, control (T3) samples grouped well in stage 2 and stage 3. In the case of *R. solani* infected soil, *Pseudomonas* (T4) and *Bacillus* (T5) showed closeness with each other in all three growth stages, and only *Rhizoctonia* control (T6) separated from others in stage 1 and 3 (Supplementary Figure 3B).

**FIGURE 4:**
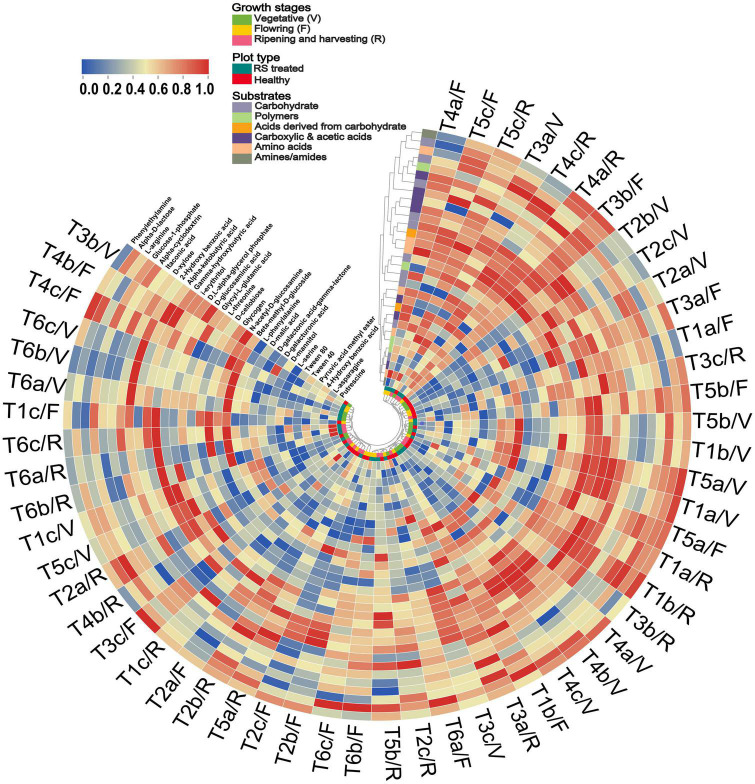
Circular heat map and hierarchical cluster analysis based on the average well color development (AWCD) at 120 h of soil microbial communities under pathogen and antagonist treated soil. Higher to low AWCD response indicated via red to blue gradient. Treatment details as Figure 2.

Additionally, the tendency of different carbon substrates between PC1 and PC2 was separated in pathogen-treated soil compared to healthy soil (Supplementary Figure 3C). In the case of healthy soil, five carbon substrates, such as C10, C16, C17, C25, and C30, showed separation from other carbon substrates (Supplementary Figure 3C). In the pathogen-treated soil case, five carbon substrates, such as C5, C7, C8, C10, C13, C15, C27, and C31, were separated from other carbon substrates (Supplementary Figure 3D). The microbial response is also represented through a circular cluster tree based on the substrate response of all treatments in different growth stages, revealing the impact of pathogen infestation on the substrate grouping and treatment clustering (Figure 4). *Pseudomonas* and *Bacillus* treatment grouped well, while *R. solani* infested soil samples separated and showed low substrate utilization response in different growth stages (Figure 4). Cluster analysis revealed that pathogen and antagonist bacteria interaction could considerably affect the community-level physiological profile.

Shannon, subtract evenness, Simpson, and the McIntosh diversity indices showed different catabolic diversity with both antagonistic bacteria during the plant growth (Supplementary Figure 4). Moreover, the highest values for all diversity parameters were recorded during stage 2 and stage 3 with all treatments (Supplementary Figure 4). The interactive effect of the independent variables for most parameters proved non-significant in healthy soil but significant with pathogen-treated soil (Table 1). Except for the Shannon diversity index, all treatments without pathogen had higher diversity indices than pathogen-treated soil (Table 1). Multiple comparisons detected a significantly higher Simpson diversity index in stage 3 with or without pathogen (Supplementary Figure 4). Likewise, the McIntosh diversity index showed a significant interaction between treatments and time. Pathogen and antagonist application induced the catabolic diversity through the substrate richness and evenness (Supplementary Figure 4). Maximum substrate richness found 31 carbon and minimum 28 substrates in the treatments.

**FIGURE 5:**
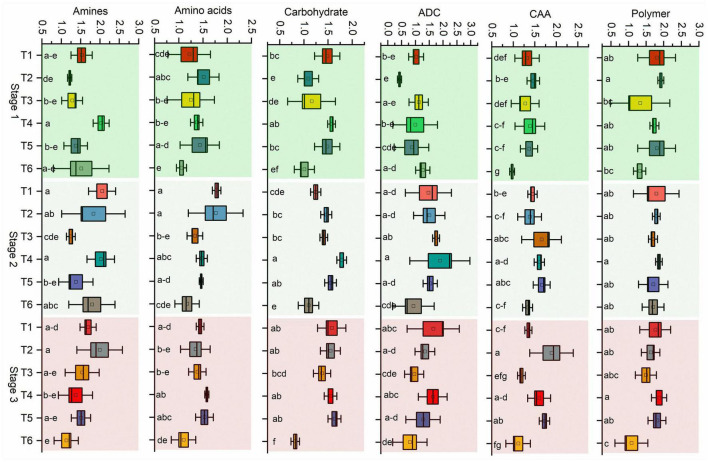
Community-level physiological profiles (CLPP) of pathogen and antagonist treated soil samples. Substrates were classified as amines/amides, amino acids, carbohydrates, Acids derived from carbohydrates (ADC), Carboxylic & acetic acids (CAA), and polymers. Mean values (*n* = 3) in the same column followed by the same letter(s) are not significantly different at (*P* < 0.05) according to the DMRT test. Treatment details as Figure 2.

Next, the microbial activity response of all substrates is categorized into six classes based on the AWCD values of all 31 substrates. *Bacillus* (T4) utilized the maximum amount of substrate amines in stage 1 and stage 2 in pathogen-treated soil, and *Pseudomonas* (T1) was used in stage 2 in healthy soil (Figure 5). The maximum rate of substrate Amino acids used by *Pseudomonas* (T1) and *Bacillus* (T2) in stage 2 in healthy soil. *Bacillus* (T4) utilized maximum concentration of substrate carbohydrate and Acids derived from carbohydrates in stage 2 in pathogen-treated soil. In the case of Carboxylic & acetic acids, a higher utilization rate resulted in *Bacillus* (T2) in the healthy soil. *Bacillus* (T4) showed a higher rate of polymer utilization in stage 2 and stage 3, and *Bacillus* (T2) was utilized in stage 1 (Figure 5). Significant effects on carbohydrate utilization were observed for antagonist treatments (*p* < 0.001) and growth stage (*p* < 0.05) and their interaction (*p* < 0.05) in pathogen-treated soil (Table 1). Two-way PERMANOVA results of all 31 substrates showed a significant effect on the microbial activity in the pathogen-treated soil samples (Table 2). In the case of carbohydrates, we observed substantial impacts on D-cellobiose, β-methyl-D-glucoside, D-xylose, and D-mannitol utilization in antagonist treatments (*p* < 0.05) and growth stage (*p* < 0.06) and their interaction (*p* < 0.05) in pathogen-treated soil (Table 2). A significant (*p* < 0.05) interactive effect of D-cellobiose (carbohydrate), D-malic acid (carboxylic & acetic acids), and L-phenylalanine (amino acids) also resulted in healthy soil samples. Moreover, substrates like D-galacturonic acid (carboxylic & acetic acids), L-asparagine (Amino acids), phenylethylamine (amines/amides), and putrescine (amines/amides) showed a significant interaction with treatments and plant growth with the pathogens (Table 2).

**TABLE 2:** The *P*-values of PERMANOVA of carbon substrates under healthy and *R. solani* infected soil during the plant development.

Carbon sources	Substrate classes	Healthy soil (-RS)			Pathogen-infected soil (+ RS)		
		T	GS	T × GS	T	GS	T × GS
Pyruvic acid methyl ester	Carbohydrate	0.589	0.156	0.006**	0.110	0.071	0.006**
Tween 40	Polymers	0.00***	0.003**	0.620	0.031*	0.084	0.689
Tween 80	Polymers	0.199	0.006**	0.701	0.805	0.189	0.396
Alpha-cyclodextrin	Polymers	0.037	0.931	0.182	0.134	0.781	0.515
Glycogen	Polymers	0.133	0.974	0.217	0.018*	0.027*	0.114
D-cellobiose	Carbohydrates	0.015*	0.007**	0.024*	0.040*	0.053	0.007**
α-D-lactose	Carbohydrates	0.424	0.117	0.595	0.454	0.252	0.114
β-methyl-D-glucoside	Carbohydrates	0.654	0.871	0.034	0.00***	0.00***	0.001**
D-xylose	Carbohydrates	0.006**	0.440	0.028*	0.00***	0.012*	0.032*
i-erythritol	Carbohydrates	0.691	0.864	0.076	0.041*	0.234	0.873
D-mannitol	Carbohydrates	0.833	0.146	0.558	0.00***	0.021*	0.00***
N-Acetyl-D-glucosamine	Carbohydrates	0.039*	0.201	0.002**	0.00***	0.505	0.040*
D-glucosaminic acid	Acids derived from carbohydrate	0.355	0.557	0.394	0.056	0.018*	0.701
Glucose-1-phosphate	Carbohydrate	0.172	0.00***	0.021*	0.451	0.223	0.004**
D,L-α-glycerol phosphate	Carbohydrate	0.038*	0.535	0.013*	0.028*	0.385	0.625
D-galactonic acid-gamma-lactone	Acids derived from carbohydrate	0.183	0.466	0.266	0.002**	0.435	0.413
D-galacturonic acid	Carboxylic & acetic acids	0.353	0.185	0.774	0.001**	0.010*	0.00***
2-Hydroxy benzoic acid	Carboxylic & acetic acids	0.668	0.001**	0.061	0.190	0.018*	0.084
4-Hydroxy benzoic acid	Carboxylic & acetic acids	0.828	0.597	0.080	0.268	0.069	0.212
γ-hydroxybutyric acid	Carboxylic & acetic acids	0.005**	0.728	0.008**	0.001**	0.055	0.126
Itaconic acid	Carboxylic & acetic acids	0.459	0.723	0.064	0.016*	0.811	0.456
α-ketobutyric acid	Carboxylic & acetic acids	0.148	0.306	0.097	0.037*	0.076	0.151
D-malic acid	Carboxylic & acetic acids	0.001**	0.00***	0.019**	0.00***	0.00***	0.751
L-arginine	Amino acids	0.578	0.024*	0.071*	0.356	0.130	0.124
L-asparagine	Amino acids	0.676	0.047*	0.295	0.072	0.015*	0.072
L-phenylalanine	Amino acids	0.012*	0.00***	0.025*	0.537	0.497	0.767
L-serine	Amino acids	0.816	0.061	0.008**	0.046*	0.172	0.054
L-threonine	Amino acids	0.294	0.611	0.050	0.047*	0.226	0.933
Glycyl-L-glutamic acid	Amino acids	0.191	0.337	0.784	0.118	0.623	0.172
Phenylethylamine	Amines/amides	0.164	0.326	0.738	0.048*	0.067	0.025*
Putrescine	Amines/amides	0.418	0.410	0.294	0.004**	0.033*	0.025*

Bacterial treatments (T): *Pseudomonas* and *Bacillus*. Growth stages (GS): different growth stages of tomato.

*p < 0.05, **p < 0.01 and ***p < 0.001.

### Correlation between soil and plant parameters with the substrates

**FIGURE 6:**
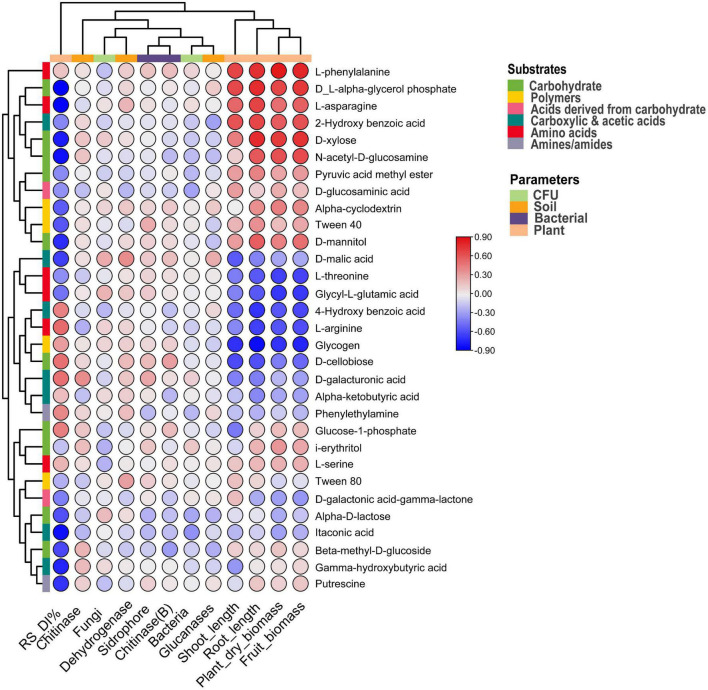
Correlation heatmap between soil and plant parameters and carbon substrates under pathogen and antagonist treated soil. Positive correlation indicated via red and negative via blue color.

The correlation between plant and soil parameters and carbon substrates is represented in Figure 6, and the p-value is indicated in Supplementary Table 1. Among the substrates, Alpha-D-lactose, D-glucosaminic acid, and itaconic acid negatively (*p* < 0.05) correlated with the bacterial CFU. A significant (*p* < 0.1) negative correlation of fungal CFU resulted with i-erythritol and L-serine, and D-malic acid showed a positive correlation (*p* < 0.01) with fungal CFU. Soil Dehydrogenase showed a positive correlation (*p* < 0.05) with Tween 80 and D-malic acid. D-galacturonic acid was positively linked with soil chitinase, and L-arginine correlated negatively. Likewise, soil glucanase negatively correlated with the substrates like Alpha-D-lactose, Beta-methyl-D-glucoside, and 2-Hydroxy benzoic acid. However, D-malic acid is positively associated with soil glucanase. In the case of bacterial siderophore, Tween 40 and D-galacturonic acid are associated positively, and Alpha-D-lactose was associated negatively. Bacterial chitinase is associated positively with D-cellobiose and associated negatively with Beta-methyl-D-glucoside. Interestingly, *Rhizoctonia* disease incidence negatively correlated (*p* < 0.05) with different substrates such as Gamma-hydroxybutyric acid, Alpha-D-lactose, Beta-methyl-D-glucoside, D-xylose, D-mannitol, N-acetyl-D-glucosamine, D-L-alpha-glycerol phosphate, itaconic acid, D-malic acid, L-asparagine, and putrescine. However, plant parameters correlated positively with different substrates like plant biomass, fruit biomass, and root length with L-phenylalanine. Fruit biomass and root length also positively correlated with the D-xylose (Figure 6 and Supplementary Table 1).

## Discussion

The rhizoplane is generally considered a hub of microbial activities that are driven by plant exudates and soil nutrients (Moe, 2013; Jones et al., 2018; Olanrewaju et al., 2019; Zhao et al., 2021). The major group of plant rhizospheric bacteria, known as plant growth-promoting rhizobacteria (PGPR), performed direct or indirect events to support plant growth through rhizosphere or endosphere colonization (Mhlongo et al., 2018; Shah et al., 2021; Vandana et al., 2021). The genus *Pseudomonas* and *Bacillus are* considered important PGPR candidates (Vocciante et al., 2022). They can aggressively colonize the rhizoplane and participate in many activities like plant growth promotion, stress tolerance, biocontrol and mineral mobilization, etc. (Solanki et al., 2012a,^2014^; Wang et al., 2020). The antagonistic bacteria (*Pseudomonas* and *Bacillus*) used in the present study significantly enhanced the plant growth in healthy and *R. solani*-treated soil (Solanki et al., 2012a,^2014^). Soil and bacterial enzymatic activities played an essential role in the biocontrol of *R. solani* (Solanki et al., 2012b; Berendsen et al., 2018; Wu et al., 2019). Soil enzymes such as dehydrogenase, chitinase, and glucanase are all hydrolytic enzymes involved in the hydrolysis and lysis of complex molecules and improves the plant systemic defense (Gurung et al., 2013; Shafi et al., 2017; Wu et al., 2019; Prasad and Raghuwanshi, 2022).

Soil dehydrogenase enzyme is used as an indicator of soil biological activity that involves the nutrients transformation between microbes and plants (Grzadziel et al., 2018; Kaur and Kaur, 2021). Chitinase and glucanase enzymes are involved in the degradation of fungal cell walls (Adams, 2004), and many hydrolytic bacteria play an essential role in disease management (Zachow et al., 2011). In the present study, the higher enzyme activity improved by applying antagonists in the pathogen-treated soil directly correlated with the disease reduction. The application of both antagonistic microbes influenced the microbial count as determined through the plate count method. Yin et al. (2021) reported that selected soil shaped the beneficial microbial communities that reduced plant root diseases and enhanced crop productivity. Specific bacterial communities played a significant role in the suppuration of *Rhizoctonia* bare patch and root rot disease of wheat (Yin et al., 2013). In the present study, the populations of bacteria and fungi in the rhizoplane soils dramatically increased after stage 1 in antagonist treatments compared to control soil samples. Conversely, fungal populations in healthy and infected soil were markedly lower in antagonist bacteria-treated soil. Bacterial densities in *Pseudomonas* and *Bacillus* treated soil were dramatically higher than those of healthy and infected control. These results demonstrate that antagonistic bacteria can significantly alter microbial community structure via propagation around the plant root zone. Zachow et al. (2011) also reported that higher numbers of bacterial groups inhibit the growth of *R. solani* in soil. Of these, only the bacterial CFU showed significant interaction in the biocontrol of *R. solani*, but the differences in fungi populations are more related to the original soil type. Based on AWCD results, the disease incidence of tomato root rot showed a negative relationship with the many substrates. It indicates that microbial substrates play an essential role in pathogen suppression. A significant negative link between antagonistic bacteria application and interaction of pathogen was observed in the study that showed the potential of antagonistic bacteria to reduce disease incidence. Several PGPR possesses antagonistic properties toward soilborne fungi, including *R. solani* (Yin et al., 2013, 2021; Solanki et al., 2014; Araujo et al., 2019). These results agree with previous studies that have shown that applications of biocontrol agents with plants positively impact soil microbial communities (Araujo et al., 2019; Huang et al., 2021). These results suggested that the application of *Bacillus* strains reduced the *R. solani* population in the soil with antifungal activity, and this action also reduced the other fungal population. Both antagonistic bacteria properly modulate the soil enzyme activity levels and effectively enhance the rhizosphere soil environment, enhancing the enzyme activities by inducing siderophore and chitinase-producing bacteria that help to improve nutrient absorption from the soil that support directly to disease resistance of the plants.

In contrast, the microbial population actively suppresses *R. solani* by competition of carbon substrate or space in the rhizosphere. The CLPP results indicated that during stage 1, at the first sampling, the microbial community response did not vary significantly in the rhizospheric soil samples. The root zone is a dynamic environment that provides nutrients like root exudates and space to shape microbial communities (Haichar et al., 2008; Edwards et al., 2015). Rhizoplane contains large numbers of diverse types of bacteria and fungi (Van Der Heijden and Schlaeppi, 2015). In the current study, antagonistic treated soil strongly affected the microbial diversity and function in healthy and infected soil, especially in the fruit development stage. The diversity indices of the pathogen-treated soil with antagonistic bacteria were higher than in healthy soil. Additionally, disease incidence was negatively related to all the diversity indexes and different carbon substrates, and plant biomass positively correlated with D-xylose and L-phenylalanine. These results indicated that the microbial communities in the pathogen-treated soil might be more robust and capable of handling competition in the presence of *R. solani*. Plants may stabilize the rhizoplane microbial community by creating a complex ecological system under the pathogen-treated soil. Compared to the pathogen-treated soil, the healthy soil exhibited the lowest level of microbial activity in stage 1 (vegetative), which then stabilized in stage 2 (flowering) and stage 3 (fruiting stage). The pathogen inoculations with antagonists treatment have dissimilarly shown an effect on the microbial activities. The microbial activity of the rhizoplane in the pathogen-treated soil was significantly higher than in healthy soil due to the substrate competition effect. These results allied with similar studies that concluded the plant stimulates the beneficial microbiome to reduce pathogen invasion and improve plant defense (Chiu et al., 2017; McLaren and Callahan, 2020). The current study indicates that antagonist microbes influenced substrate utilization strongly in stage 3 (fruit development stage) in healthy soil. In the case of the pathogen-treated land, growth stages-based fluctuations have been observed with soil enzymes and microbial function as well as substrate diversity indices. Correlation results provide the significance of different substrates in the biocontrol of pathogens. Plant, soil, and CLPP parameter provide insight into the role of carbon substrates in pathogen suppuration during plant growth. The carboxylic acid that significantly contributed to the control of *R. solani* was pyruvic acid methyl ester, an intermediate of the citric acid cycle (Frolkis et al., 2010).

Carbohydrates that had a significant interaction with the biocontrol of *R. solani* were β-methyl-D-glucoside, D-mannitol, and N-acetyl-D-glucosamine. These carbohydrates played an essential role in microbial growth in the plant rhizosphere (Adams et al., 2017; Weng et al., 2022). N-acetyl-D-glucosamine is a significant component of *R. solani* call wall (Benyagoub et al., 1996). D-galacturonic acid (carboxylic & acetic acids) that had a significant interactive effect in biocontrol is also known as the backbone of plants’ mechanical strength (Hongo et al., 2012). L-asparagine (amino acids) and amines/amides (phenylalanine and putrescine) are the essential nutrients for microbial growth in the plant rhizosphere (Haichar et al., 2008; Adams et al., 2017; Weng et al., 2022).

In conclusion, carbohydrates, carboxylic & acetic acids, amino acids, and amines/amides are the major key player in rhizospheric biology in the presence of the pathogen. It showed a discernible variation in the rhizoplane communities’ function with pathogen-treated and healthy soil. A significant shift of microbial function protects the plant from the pathogen in different growth stages, and microbial substrate utilization pattern is induced in the fruiting and ripening stage with antagonists. Current study results answered that the substrate-based mechanism study of pathogenic and healthy soil might generate meaningful information that can help to shape or modify the microbial community to improve the plant disease management system. However, an in-depth analysis is needed in the future to understand microbial association in root pathogenesis, especially microbial transformation, recruitment, and complex functional mechanism in microbes-microbes interaction. It can be concluded that the BIOLOG based EcoPlate method resulted am useful tool to study the variability of the potential antagonist and pathogen, as significant variation have been obtained. Additionally, the results obtained from the EcoPlate analysis correlate with the pathogen reduction and plant growth stimulation that signifies the current study and this method can be an excellent tools for the study of pathogen antagonist, plant-microbes and other interactive filed that have substrate played the important role.

## Data availability statement

The raw data supporting the conclusions of this article will be made available by the authors, without undue reservation.

## Author contributions

MS, SK, MY, PK, and AKS contributed to conception and design of the study. MS, SS, and SR performed the experiments. BK, PD, and SKh performed the statistical analysis. MS, ACS, PK, and MY wrote the first draft of the manuscript. SK, AKS, ACS, BA, MJ, and KQ revised and finalized the manuscript. All authors contributed to manuscript revision, read, and approved the submitted version.
