# Supplementary material for: Functional interplay between antagonistic bacteria and Rhizoctonia solani in the tomato plant rhizosphere
Source: Front Microbiol. 2022 Sep 26;13:990850. doi: 10.3389/fmicb.2022.990850 (PMC9548980; doi:10.3389/fmicb.2022.990850)
Supplement: Supplementary file 1 [file Table_1.DOCX]

**
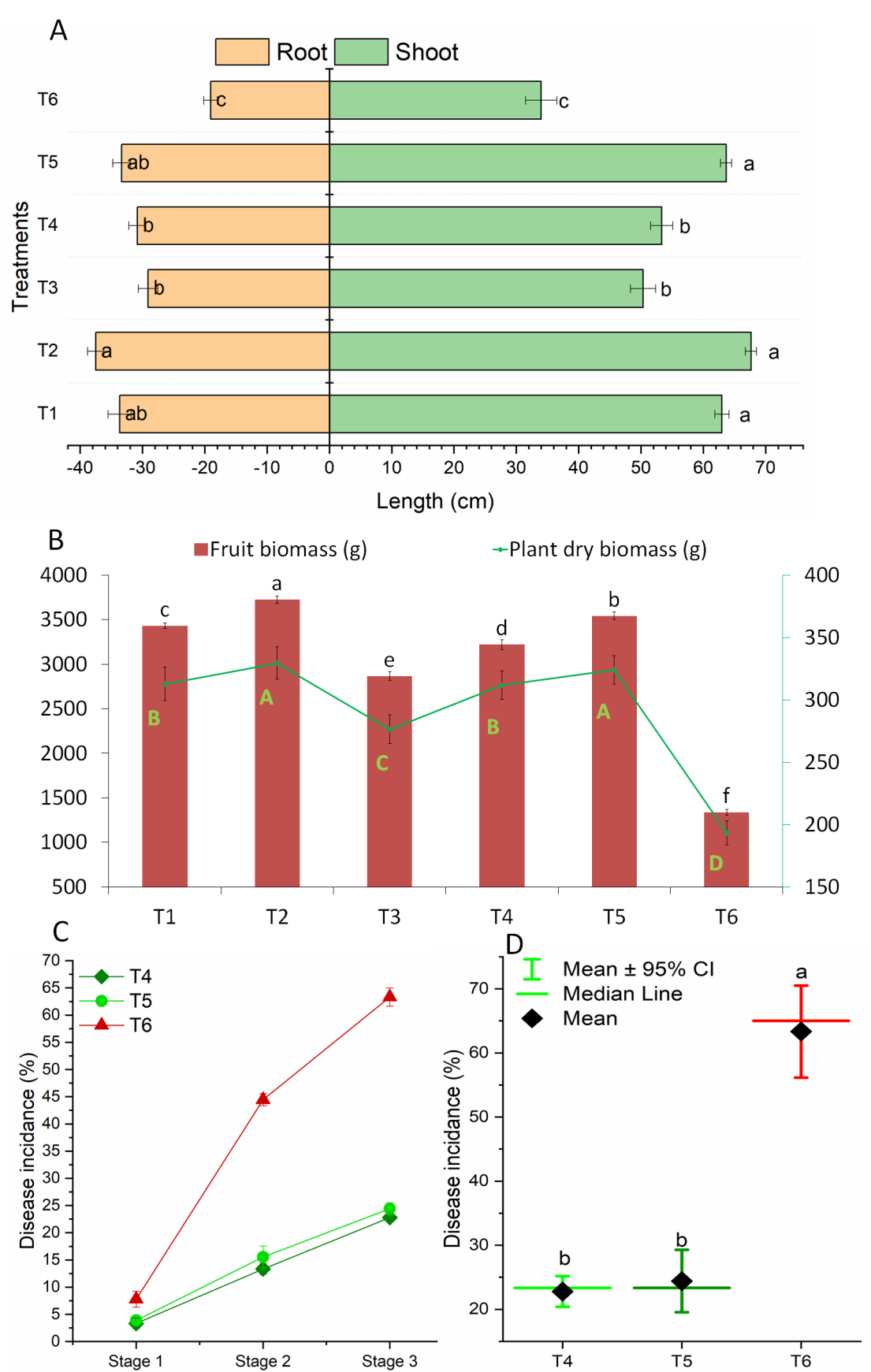
**

**Figure S1**: Plant physiological parameter after harvesting with or without *R. solani* under field conditions. Plant root and shoot length (A); Plant and fruit biomass (B); (C) *R. solani* disease incidence during the plant growth; (D) *R. solani* disease incidence at stage 3. Treatments: (T1) *Pseudomonas* alone, (T2) *Bacillus* alone, (T3) healthy control (autoclaved liquid suspension without bacteria), (T4) antagonist *Pseudomonas* + *R. solani*, (T5) *Bacillus* + *R. solani*, and (T6) *R. solani* alone with autoclaved liquid suspension without bacteria. Stage 1(vegetative stage), Stage 2 (flowering stage), and Stage 3 (fruit ripening stage). Mean values (n=3) in the same column followed by the same letter(s) are not significantly different at (*P*<0.05) according to the DMRT test.

**
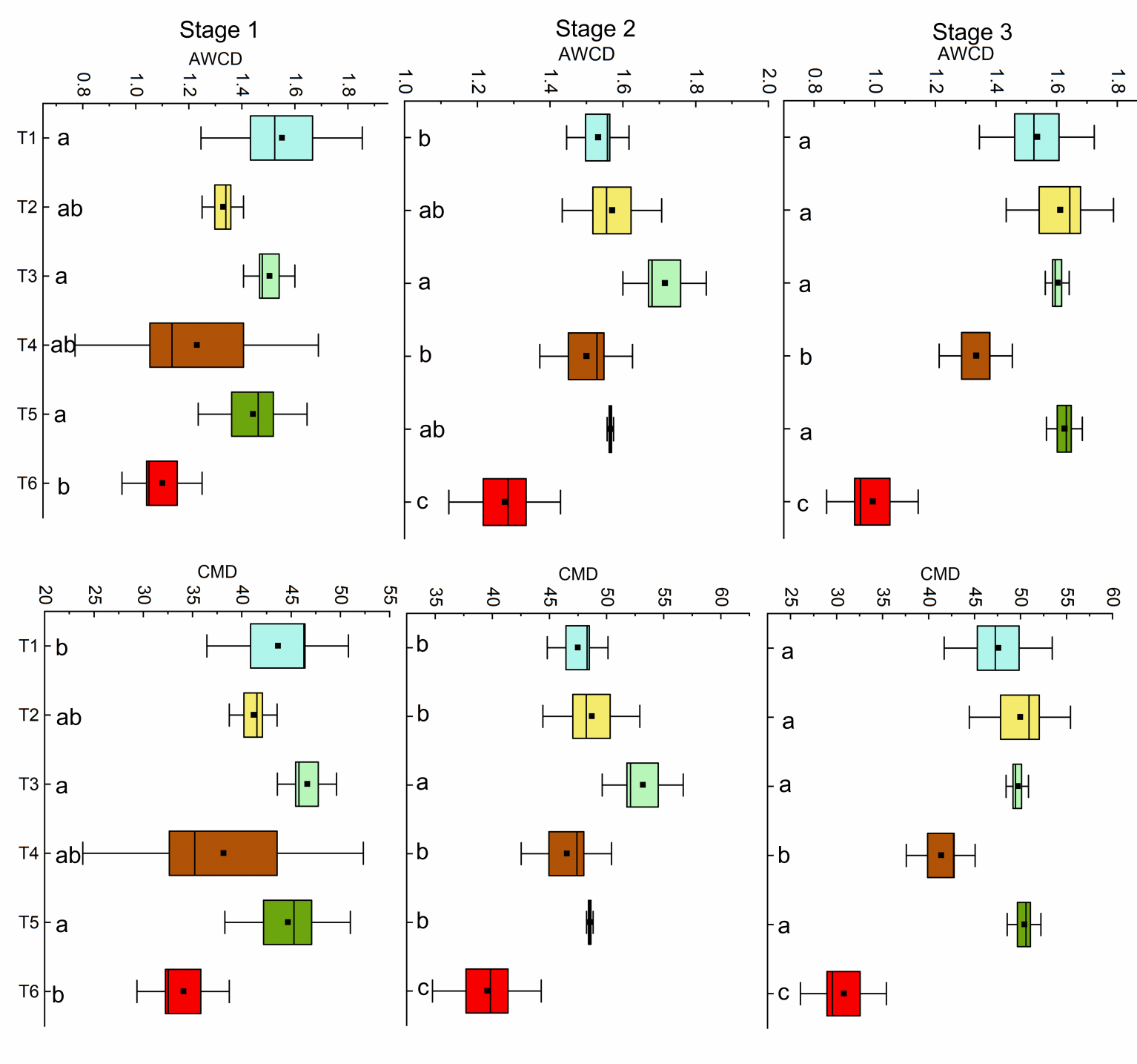
Figure S2:** Average well color development (AWCD) and community metabolic diversity (CMD) of pathogen and antagonist treated soil of tomato plant. Treatments: (T1) *Pseudomonas* alone, (T2) *Bacillus* alone, (T3) healthy control (autoclaved liquid suspension without bacteria), (T4) antagonist *Pseudomonas* + *R. solani*, (T5) *Bacillus* + *R. solani*, and (T6) *R. solani* alone with autoclaved liquid suspension without bacteria. Stage 1(vegetative stage), Stage 2 (flowering stage), and Stage 3 (fruit ripening stage). Mean values (n=3) in the same column followed by the same letter(s) are not significantly different at (*P*<0.05) according to the DMRT test.


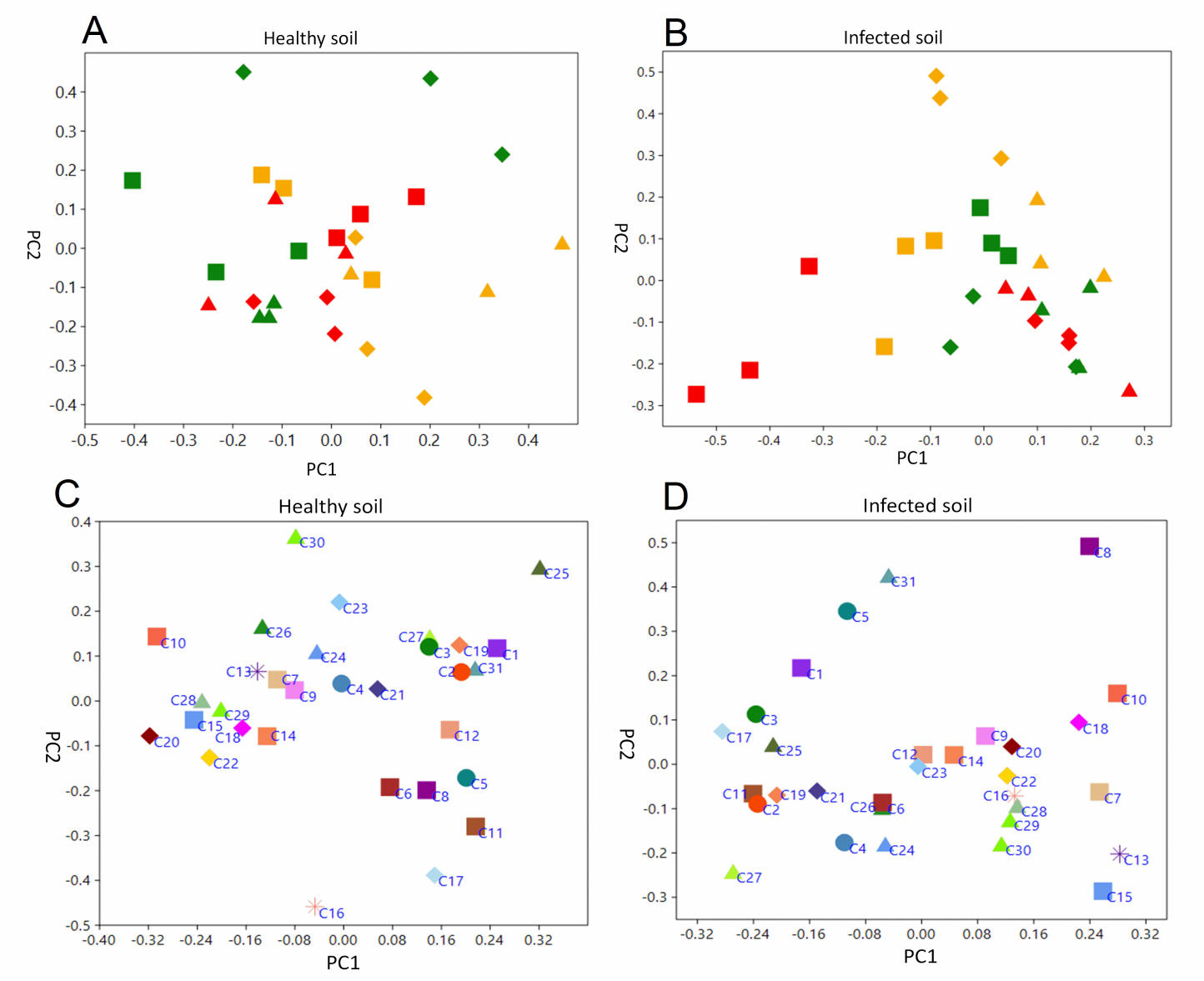


**Figure S3**: PCA of the CLPP response of pathogen and antagonist treated soil of tomato plant. Healthy soil (A) and Pathogen-treated soil. Treatments for **A and B**: T1 & T4 (**diamond shape**), T2 & T5 (**tringle shape**), T3 & T6 (**square shape**); stage 1 (**orange color**), stage 2(**green color**), and stage 3(**red color**). Treatment details as **figure S2**. Carbon substrate for C and D: C1-Pyruvic acid methyl ester, C2-Tween 40, C3-Tween 80, C4-Alpha-cyclodextrin, C5-Glycogen, C6-D-cellobiose, C7-Alpha-D-lactose, C8-Beta-methyl-D-glucoside, C9-D-xylose, C10-i-erythritol, C11-D-mannitol, C12-N-acetyl-D-glucosamine, C13-D-glucosaminic acid, C14-Glucose-1-phosphate, C15-D,L-alpha-glycerol phosphate, C16-D-galactonic acid-gamma-lactone, C17-D-galacturonic acid, C18-2-Hydroxy benzoic acid, C19-4-Hydroxy benzoic acid, C20-Gamma-hydroxybutyric acid, C21-Itaconic acid, C22-Alpha-ketobutyric acid, C23-D-malic acid, C24-L-arginine, C25-L-asparagine, C26-L-phenylalanine, C27-L-serine, C28-L-threonine, C29-Glycyl-L-glutamic acid, C30-Phenylethylamine, C31-Putrescine.

**
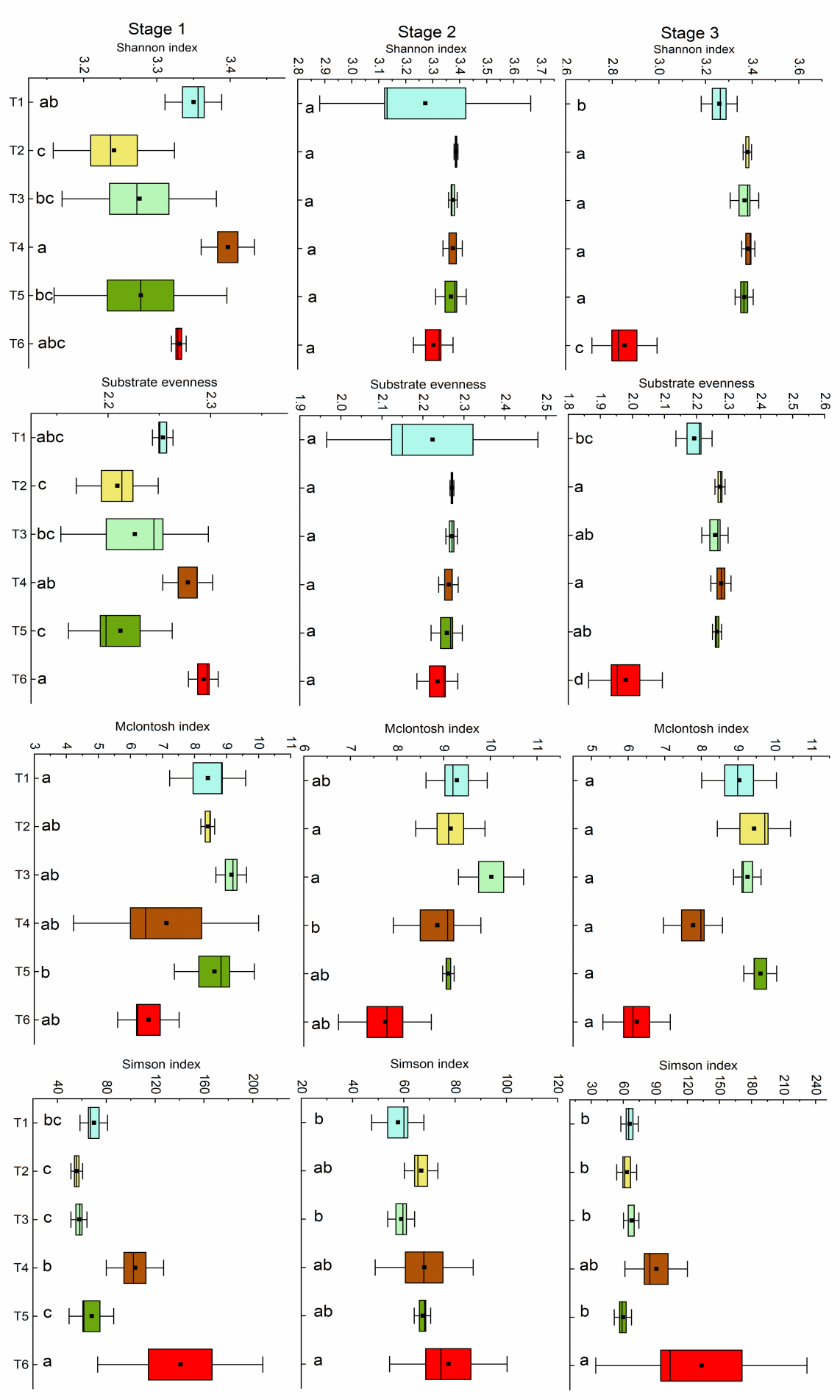
**

**Figure S4**: Microbial functional diversity indexes of pathogen and antagonist treated soil of tomato plant. Mean values (n=3) in the same column followed by the same letter(s) are not significantly different at (*P*<0.05) according to the DMRT test.

Table S1: *P* values of correlation analysis between soil and plant parameters with carbon substrates.

|  | CFU | | Soil enzymes | | | Bacterial | | Plant parameters | | | | |
| --- | --- | --- | --- | --- | --- | --- | --- | --- | --- | --- | --- | --- |
| Substrates | CFU_Bacteria | CFU_Fungi | Soil_Dehydrogenaseee | Soil_Chitinase | Soil_Glucanase | Siderophore | Chitinase | RS_DI% | PDB | FB | RL | SL |
| Pyruvic acid methyl ester | 0.13 | 0.33 | 0.35 | 0.82 | 0.53 | 0.58 | 0.98 | 0.31 | 0.59 | 0.53 | 0.41 | 0.56 |
| Tween 40 | 0.95 | 0.68 | 0.49 | 0.72 | 0.31 | 0.07 | 0.67 | 0.11 | 0.74 | 0.62 | 0.49 | 0.70 |
| Tween 80 | 0.69 | 0.88 | 0.03 | 0.23 | 0.89 | 0.41 | 0.81 | 0.53 | 0.83 | 0.88 | 0.88 | 0.76 |
| Alpha-cyclodextrin | 0.99 | 0.55 | 0.33 | 0.91 | 0.36 | 0.54 | 0.38 | 0.13 | 0.39 | 0.44 | 0.48 | 0.97 |
| Glycogen | 0.52 | 0.56 | 0.47 | 0.87 | 0.31 | 0.61 | 0.46 | 0.42 | 0.12 | 0.08 | 0.03 | 0.11 |
| D-cellobiose | 0.67 | 0.68 | 0.18 | 0.61 | 0.66 | 0.18 | 0.03 | 0.19 | 0.40 | 0.31 | 0.22 | 0.19 |
| Alpha-D-lactose | 0.04 | 0.19 | 0.68 | 0.23 | 0.09 | 0.09 | 0.17 | 0.10 | 0.61 | 0.75 | 0.91 | 0.88 |
| Beta-methyl-D-glucoside | 0.31 | 0.48 | 0.24 | 0.12 | 0.07 | 0.30 | 0.02 | 0.07 | 0.79 | 0.89 | 0.89 | 0.84 |
| D-xylose | 0.26 | 0.37 | 0.76 | 0.32 | 0.31 | 0.84 | 0.46 | 0.01 | 0.11 | 0.09 | 0.07 | 0.38 |
| i-erythritol | 0.33 | 0.08 | 0.85 | 0.21 | 0.96 | 0.33 | 0.62 | 0.64 | 0.52 | 0.61 | 0.69 | 0.84 |
| D-mannitol | 0.62 | 0.59 | 0.63 | 0.75 | 0.20 | 0.50 | 0.64 | 0.03 | 0.41 | 0.32 | 0.25 | 0.55 |
| N-acetyl-D-glucosamine | 0.14 | 0.57 | 0.55 | 0.28 | 0.15 | 0.67 | 0.14 | 0.01 | 0.19 | 0.17 | 0.22 | 0.84 |
| D-glucosaminic acid | 0.03 | 0.87 | 0.12 | 0.16 | 0.92 | 0.46 | 0.31 | 0.34 | 0.65 | 0.75 | 0.84 | 0.55 |
| Glucose-1-phosphate | 0.68 | 0.21 | 0.49 | 0.30 | 0.37 | 0.77 | 0.20 | 0.25 | 0.74 | 0.73 | 0.86 | 0.38 |
| D,L-alpha-glycerol phosphate | 0.66 | 0.53 | 0.57 | 0.88 | 0.38 | 0.79 | 0.92 | 0.00 | 0.15 | 0.11 | 0.07 | 0.16 |
| D-galactonic acid-gamma-lactone | 0.87 | 0.71 | 0.29 | 0.69 | 0.86 | 0.91 | 0.24 | 0.25 | 0.55 | 0.51 | 0.62 | 0.73 |
| D-galacturonic acid | 0.46 | 0.38 | 0.33 | 0.01 | 0.80 | 0.05 | 0.75 | 0.19 | 0.68 | 0.56 | 0.41 | 0.41 |
| 2-Hydroxy benzoic acid | 0.24 | 0.34 | 0.61 | 0.57 | 0.03 | 0.62 | 0.59 | 0.29 | 0.23 | 0.20 | 0.15 | 0.20 |
| 4-Hydroxy benzoic acid | 0.71 | 0.12 | 0.62 | 0.48 | 0.61 | 0.84 | 0.20 | 0.26 | 0.23 | 0.19 | 0.12 | 0.32 |
| Gamma-hydroxybutyric acid | 0.42 | 0.64 | 0.97 | 0.17 | 0.36 | 0.85 | 0.92 | 0.02 | 0.95 | 0.88 | 0.99 | 0.51 |
| Itaconic acid | 0.01 | 0.88 | 0.40 | 0.11 | 0.46 | 0.11 | 0.16 | 0.00 | 0.56 | 0.64 | 0.80 | 0.67 |
| Alpha-ketobutyric acid | 0.96 | 0.63 | 0.43 | 0.18 | 0.59 | 0.87 | 0.12 | 0.66 | 0.59 | 0.57 | 0.44 | 0.73 |
| D-malic acid | 0.98 | 0.07 | 0.01 | 0.75 | 0.08 | 0.37 | 0.24 | 0.05 | 0.59 | 0.60 | 0.43 | 0.25 |
| L-arginine | 0.30 | 0.52 | 0.55 | 0.07 | 0.51 | 0.77 | 0.35 | 0.15 | 0.23 | 0.21 | 0.16 | 0.44 |
| L-asparagine | 0.72 | 0.82 | 0.14 | 0.49 | 0.94 | 0.77 | 0.67 | 0.00 | 0.32 | 0.25 | 0.14 | 0.28 |
| L-phenylalanine | 0.55 | 0.18 | 0.42 | 0.80 | 0.99 | 0.27 | 0.22 | 0.70 | 0.03 | 0.06 | 0.08 | 0.15 |
| L-serine | 0.97 | 0.09 | 0.98 | 0.71 | 0.84 | 0.79 | 0.76 | 0.59 | 0.67 | 0.66 | 0.71 | 0.79 |
| L-threonine | 0.97 | 0.68 | 0.85 | 0.25 | 0.97 | 0.54 | 0.74 | 0.33 | 0.17 | 0.18 | 0.25 | 0.39 |
| Glycyl-L-glutamic acid | 0.82 | 0.12 | 0.33 | 0.95 | 0.94 | 0.37 | 0.89 | 0.21 | 0.13 | 0.14 | 0.25 | 0.42 |
| Phenylethylamine | 0.11 | 1.00 | 0.22 | 0.55 | 0.56 | 0.13 | 0.74 | 0.29 | 0.78 | 0.68 | 0.65 | 0.72 |
| Putrescine | 0.81 | 0.17 | 0.48 | 0.46 | 0.80 | 0.48 | 0.94 | 0.04 | 0.85 | 0.79 | 0.79 | 0.87 |
| Cell forming unit (CFU), *Rhizoctonia solani* (RS), Plant dry biomass (PDB), Fresh biomass (FB), Root length (RL), Shoot length (SL) | | | | | | | | | | | | |
